# The Therapeutic Effects of 4-Aminopyridine Administration via Phonophoresis on Sutured Postoperative Wounds: A Histopathological, Immunohistochemical, Molecular, and Morphometric Study

**DOI:** 10.3390/biomedicines14102393

**Published:** 2026-10-19

**Authors:** Selma Yaman, Hamit Yilmaz, Gamze Altun, Belemir Gülhan, Mustafa Cicek, Levent Tumkaya

**Affiliations:** 1 Department of Biophysics, Faculty of Medicine, Kahramanmaras Sutcu Imam University, Kahramanmaras 46040, Türkiye; 2 Department of Histology & Embryology, Faculty of Medicine, Ondokuz Mayıs University, Samsun 55200, Türkiyelevent.tumkaya@omu.edu.tr (L.T.); 3 Department of Histology & Embryology, Faculty of Medicine, Samsun University, Samsun 55080, Türkiye; belemir.gulhan@samsun.edu.tr; 4 Department of Medical Biology, Faculty of Medicine, Kahramanmaras Sutcu Imam University, Kahramanmaras 46040, Türkiye

**Keywords:** 4-aminopyridine, ultrasound, phonophoresis, VEGFR, MMP9, EGFR, MMP2, TGF-β

## Abstract

**Background**: The skin is a crucial barrier between the body and the external environment; however, its natural healing ability may be compromised in deep injuries. This study aimed to explore the topical therapeutic effects of phonophoresis of 4-aminopyridine (4-AP, a potassium (K+) channel blocker) applied to sutured wound tissue using ultrasound (US/phonophoresis). **Methods**: This study utilized phonophoresis to enhance the transdermal delivery of 4-AP in 49 male Sprague-Dawley rats. An incision was created in the right facial area of each rat and closed with four separate sutures. The animals were randomly assigned to seven groups (n = 7): Control, Sham, US, 4-AP, and three groups receiving different doses of 4-AP (Low (L), Medium (M), and High (H)) + US. 4-AP was administered topically via the transdermal route in a hydrogel with DMSO. **Results**: The results showed that 4-AP alone was not sufficiently effective, but the 4-AP + US group showed a 17.9-fold increase in EGFR expression and a 26.2-fold increase in VEGFR expression. The increase in MMP9 activity (28-fold) was in line with the immunohistochemical findings and morphological measurements. Overall, the medium dose appeared to offer a better balance between the rate and quality of recovery. **Conclusions**: This study presents a regenerative treatment strategy with promising therapeutic effects, promoting tissue restoration through the topical effects of phonophoresis on angiogenesis, proliferation, and tissue remodeling in surgically sutured wounds via US-induced cavitation and 4-AP-mediated ion channel modulation.

## 1. Introduction

The skin, which covers a large surface area of the body, is a vital biological system with functions such as maintaining homeostasis, thermoregulation, and acting as a protective barrier against the external environment [1]. Disruption of the protective barrier and the resulting loss of epidermal integrity initiate a complex regenerative process in the organism that requires a high degree of coordination at the cellular and molecular levels; this process involves hemostasis, inflammation, proliferation, and remodeling [1,2].

In more serious clinical situations where natural healing mechanisms are compromised, such as deep excisions, severe burns, and diabetic ulcers, the process often leads to pathological scar tissue [1]. Recent studies have shown that 4-aminopyridine (4-AP), a selective potassium channel blocker, plays a key role in skin regeneration and cutaneous nerve repair processes, particularly targeting K^+^ channels, specifically the Kv1.3 and Kv7.2/7.3 subtypes [3]. This agent, frequently used in the treatment of multiple sclerosis (MS) to restore nerve transmission in demyelinated axons, has also been reported in several studies to exert its reparative effect by triggering a regenerative cascade through ionic mechanisms in skin tissue [4,5]. The pharmacological action of 4-AP is primarily related to the blockade of voltage-gated potassium channels, which reduces outward K^+^ current and may prolong membrane depolarization. This change can indirectly influence the activation of voltage-gated calcium channels and downstream calcium-dependent signaling pathways [5]. Subsequently, this can lead to the opening of voltage-gated calcium (Ca^2+^) channels and an increase in intracellular free calcium concentration, which in turn can regulate gene transcription through associated signaling proteins such as calmodulin [6]. This calcium-dependent signaling pathway directly stimulates the synthesis of neurotrophic factors involved in tissue repair, such as nerve growth factor (NGF) and brain-derived neurotrophic factor (BDNF) [3,4]. However, the transdermal delivery of specific molecules such as 4-AP is limited by the barrier function of the stratum corneum (SC), the outermost layer of the skin, which restricts penetration through the skin [7]. At this point, phonophoresis, a method that utilizes the biophysical effects of US waves, stands out as a promising approach [8,9]. Phonophoresis is a method of delivering active drugs that can reversibly increase skin permeability by using the mechanical and thermal effects generated by US waves, and its therapeutic efficacy has been documented [10,11,12]. The basic mechanism of this method is based on cavitation and cavitation dynamics, defined as the formation of microscopic gas bubbles in tissues due to the negative pressure generated by US waves. It has been reported that US waves, particularly in the low-frequency range (20–150 kHz), increase skin permeability and substance diffusion more effectively than high-frequency waves because they allow more time for gas bubbles to grow [13,14]. Another question to be addressed at this point is how these cavitation events occurring within the SC contribute to the process. Evidence explaining this shows that the organization of keratinocytes and the lipid bilayer is disrupted, creating temporary “aqueous channels” in the lipid lamellae, thus significantly increasing the passive diffusion rate of molecules [15].

Furthermore, evidence suggests that US-induced acoustic flux enhances drug penetration by directing drug molecules through existing pathways, such as hair follicles and sweat glands [3]. It has been reported that dual-frequency systems increase permeability up to 51 times through these pathways [13]. These and similar biophysical application processes allow for the direct delivery of active molecules such as 4-AP to the wound site in high concentrations, while minimizing the risk of systemic toxicity such as seizures and nausea, which are unwanted effects of oral and other routes of drug administration [16]. Results from experimental models indicate that phonophoresis-mediated 4-AP application increases the wound closure rate by approximately 25% compared with the control group, with statistically significant differences observed from the third postoperative day onward [3,17]. In addition to these findings, it has been shown to improve the rate and quality of healing. It promotes angiogenesis through increased VEGF and eNOS expression, accelerates macrophage polarization by suppressing pro-inflammatory cytokines (IL-1β, TNFα), increases the expression of proteins such as Keratin-14, and supports functional re-epithelialization. Furthermore, within a holistic restoration framework, 4-AP has been shown to regulate cutaneous neurotissue interactions by enhancing p75-NTR and SOX10 expression through the reparative capacity of Schwann cells [4].

In conclusion, the phonophoresis-mediated 4-aminopyridine strategy may represent a promising therapeutic option for biophysical and pharmacological synergy in regenerative medicine. This study aimed to comprehensively evaluate the topical therapeutic effects of ultrasound cavitation-mediated dermal penetration and 4-AP ion modulation on inflammation control, angiogenesis, collagen synthesis, and functional tissue restoration during sutured wound healing following surgical procedures, using immunohistochemical, molecular, and morphological analyses.

## 2. Materials and Methods

### 2.1. Experimental Animals and Ethical Approval

In this study, 49 male Sprague-Dawley rats (2–3 months old) obtained from the Kahramanmaraş Sütçü İmam University Experimental Research and Application Center were used. All experimental procedures were carried out in accordance with the National Institutes of Health Guidelines for the Care and Use of Laboratory Animals (NIH Guidelines for the Care and Use of Laboratory Animals) and were approved by the KSÜ Animal Experiments Ethics Committee (Approval No. 2025/01, Decision No. 03, 30 January 2025). The animals were housed in standard cages and provided with unlimited access to food and water. Housing conditions were maintained under a constant ambient temperature of 22–25 °C and 40–60% relative humidity with a 12/12-h light–dark cycle. The general health status of the animals was monitored daily throughout the experiment.

### 2.2. Anesthesia Protocol

Before all surgical procedures, rats were anesthetized with a combination of ketamine hydrochloride and xylazine hydrochloride (Rompun^®^, Bayer, Germany). Ketamine was administered intraperitoneally (i.p.) at a dose of 50 mg/kg, and xylazine at a dose of 10 mg/kg. The depth of anesthesia was confirmed by monitoring the leg withdrawal reflex and respiratory pattern.

### 2.3. Surgical Skin Incision and Suturing Procedure

A linear skin incision of standard length (≈2 cm) was made in the right facial region. The incision was limited to the epidermis and dermis, with minimal disruption to the subcutaneous tissues. The incision was primarily closed with four simple, separate sutures placed at equal intervals using non-absorbable natural silk surgical suture material 5-0, Doğsan, Trabzon, Turkey). Each suture was placed approximately 2–3 mm away from the wound edges, with a distance of 3–4 mm between sutures. Suturing was performed with care to minimize tissue tension and trauma while ensuring adequate wound margin approximation. Given the high mobility of the facial region, non-absorbable suture material was chosen to maintain wound margin stability.

All surgical procedures were performed by the same researcher using the same technique, and operator-related variations were minimized. Postoperative wound sites were monitored daily for infection, dehiscence, and abnormal inflammation. The facial region was selected because it represents a mechanically active and highly vascularized skin area containing abundant hair follicles and is subjected to repeated movement-related tension after suturing. This site therefore allowed the treatment response to be evaluated under a relatively challenging wound healing condition. The limited size of the tissue obtained from the facial region restricted the application of subsequent biomechanical tests, which was acknowledged as a limitation of this study.

#### 2.3.1. Experimental Design and Treatment Groups

The rats were randomly divided into seven experimental groups, with seven animals in each group. In the control group, no surgical procedure or treatment was performed on the animals. In the Sham group, only skin incision and suturing were performed; no additional treatment was applied. In the ultrasound group (US), ultrasound was applied to the affected area once daily for two weeks after suturing. In the transdermal 4-AP group (4-AP), a topical hydrogel formulation containing 7.5 µL of 4-aminopyridine and prepared with 20 mg/mL DMSO was applied to the suture line daily for two weeks. The 4-AP used in this study is a K^+^ channel blocker and was obtained from Abcam (ab120122, 100 mg; lot no. APN12470-1-1; Abcam, Cambridge, UK). In the phonophoresis groups, ultrasound (US) was applied following transdermal 4-AP application. In the 4-AP(L) + US group, a hydrogel formulation containing 5 µL of 4-AP + 13.5 mg/mL DMSO was applied to the relevant area; in the 4-AP(M) + US group, a hydrogel formulation containing 7.5 µL of 4-AP + 20 mg/mL DMSO was applied; and in the 4-AP(H) + US group, a hydrogel formulation containing 10 µL of 4-AP + 26.5 mg/mL DMSO was applied. Ultrasound treatment was performed after each application. In all experimental groups, treatments were applied daily for two weeks.

#### 2.3.2. Ultrasound and Phonophoresis Application

US/phonophoresis applications were performed using the modified BTL SMART 1 ultrasound device (BTL Industries Ltd., Hertfordshire, UK). To minimize inter-individual variation, the same researcher performed all applications. A 1 cm^2^ probe head was used at an intensity of 3 W/cm^2^ for 2 min, with circular movements over the relevant area [18].

#### 2.3.3. Chemical Reagents and Formulation Groups

4-AP was obtained from Abcam (Sigma-Aldrich, St. Louis, MO, USA; lot no.: APN12470-1-1). For transdermal applications, 4-AP was dissolved in a hydrophilic gel matrix containing DMSO and applied daily as hydrogel formulation [3,4].

### 2.4. Histopathological Applications

Dissected tissues were fixed in a mixture of 4% paraformaldehyde and 4% glutaraldehyde and then embedded in paraffin blocks after routine tissue processing. Paraffin sections (5 µm) from tissues of animals in each group were cut using a microtome, kept overnight in an oven at 58 °C, and then deparaffinized with a xylene series. The sections, dehydrated by passing them through an alcohol series, were then stained with hematoxylin and eosin. Images were obtained at different magnifications using a light microscope with a camera attachment (Olympus, BX43, Center Valley, PA, USA) and histopathologically evaluated. Immunohistochemical staining was also performed on 4 µm-thick sections from each animal. In staining performed using the HRP/AEC chromogen kit, anti-VEGF (Sc7269, Santa Cruz Biotechnology, Dallas, TX, USA), anti-TGF-β1 (sc130348, Santa Cruz Biotechnology, Dallas, TX, USA), and anti-PCNA (sc56, Santa Cruz Biotechnology, Dallas, TX, USA) were used as primary antibodies. Counterstaining was performed with Mayer’s hematoxylin. In immunohistochemical scoring, staining intensity was evaluated as 0 (none), 1 (slight), 2 (moderate), and 3 (severe).

### 2.5. Biomolecular Applications

#### Determination of Gene Expression Levels

Total RNA was isolated from tissue samples obtained from all experimental groups following the termination of the study, using a commercial RNA isolation kit (Magen Biotechnology (Guangzhou) Co., Ltd., Guangzhou, China) according to the manufacturer’s recommendations. The concentrations and purity of the RNA samples were evaluated by measuring the absorbance values at 260 and 280 nm using a spectrophotometric method. RNA samples with a suitable A260/A280 ratio were included in the study.

For complementary DNA (cDNA) synthesis, a reverse transcription kit was used according to the manufacturer’s protocol. Quantitative real-time polymerase chain reaction (qRT-PCR) analyses were performed using an Applied Biosystems 7500 Fast Real-Time PCR System. PCR reactions were prepared in a total volume of 20 µL using cDNA samples, gene-specific forward and reverse primers, SYBR Green Master Mix, and nuclease-free water, and all samples were analyzed in triplicate. The PCR amplification protocol consisted of 10 min of incubation at 95 °C, followed by 40 cycles of denaturation at 95 °C for 15 s and annealing/extension at 60 °C for 1 min. Melt curve analysis was performed to verify the specificity of the amplified products, and primer dimer formation was evaluated to assess non-specific amplification.

In this study, the expression levels of epidermal growth factor receptor (EGFR), vascular endothelial growth factor receptor (VEGFR), transforming growth factor beta (TGF-β), matrix metalloproteinase-2 (MMP2), and matrix metalloproteinase-9 (MMP9) genes were examined. The GAPDH gene was used as an internal control (housekeeping gene). Relative gene expression levels were calculated using the comparative Ct method (2^−ΔΔCt^ method). First, ΔCt values were obtained by subtracting the GAPDH Ct values from the target gene Ct values. Then, ΔΔCt values calculated using the Sham group as the calibrator group. Relative gene expression levels were expressed as fold change using the following formula: Fold Change = 2^−ΔΔCt^ (Table 1).

### 2.6. Morphometric Evaluation

The method used for the morphological evaluation of the wound area in this study was as follows: According to the simple ruler method, the surface area was determined by multiplying the length and width of the wound. To assess the third dimension of the wound, the wound thickness was measured from the isolated skin using an electronic caliper. These measurements were used to determine the morphological characteristics of the wounds based on mass, area, and volume [19].

### 2.7. Statistical Analysis

All statistical analyses were performed using GraphPad Prism (version 10.2) software. Data were expressed as the mean ± standard deviation (mean ± SD). The normality of the data distribution was assessed using the Shapiro–Wilk test. For normally distributed data, intergroup comparisons were performed using one-way analysis of variance (one-way ANOVA), followed by Tukey’s multiple comparison post hoc test to determine significant differences. For data that did not follow a normal distribution, the Kruskal–Wallis test was applied, followed by Dunn’s post hoc test for pairwise group comparisons. In the relative gene expression analyses obtained by qRT-PCR, the Sham group was considered the calibrator group. The statistical significance level was set at *p* < 0.05, while *p* < 0.01 and *p* < 0.001 were considered statistically significant.

## 3. Results

### 3.1. Histopathological Findings

**Figure 1:**
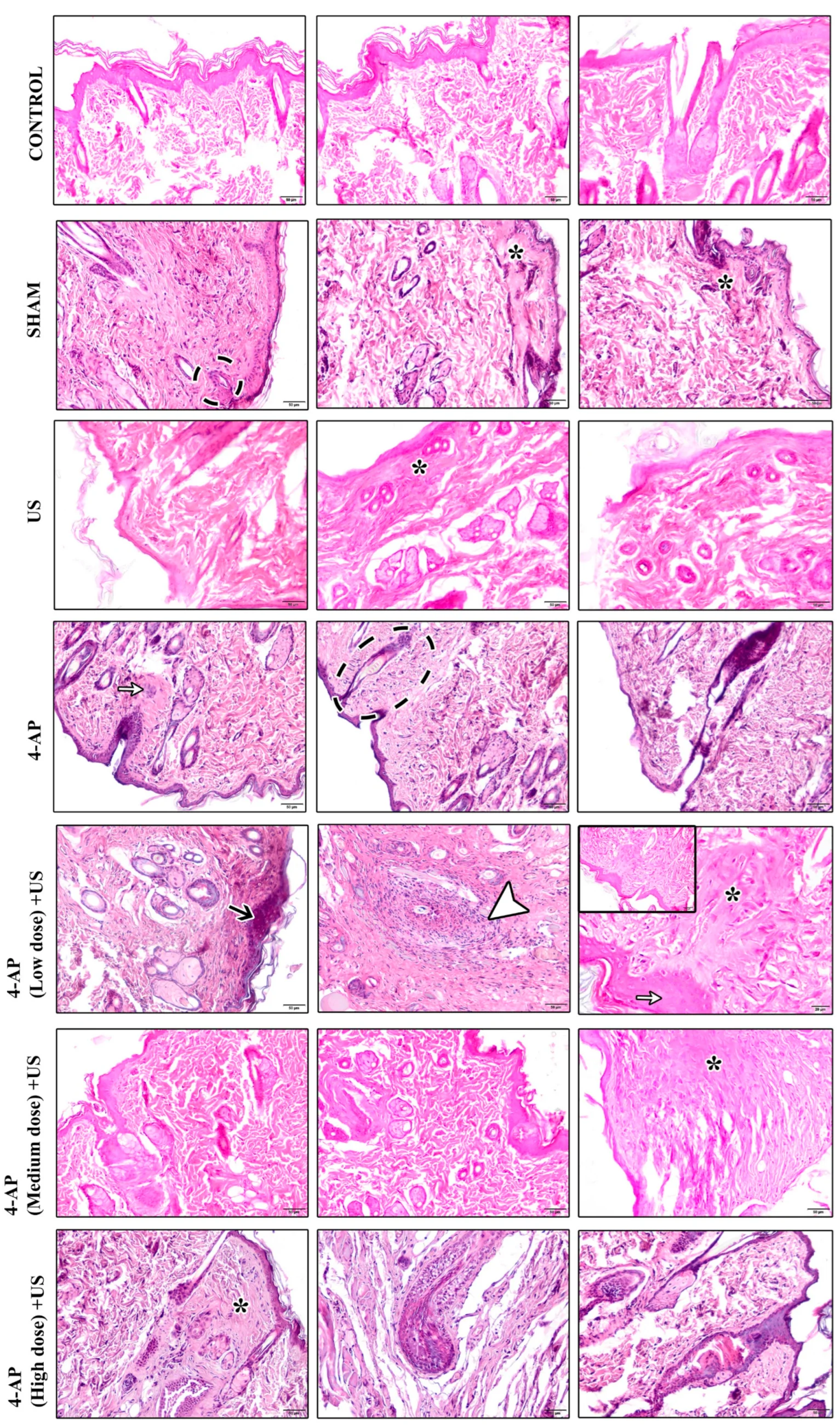
Light microscopic images of all groups are shown. In the control group, the epidermis and dermis layers appear healthy. No inflammatory cells or pathological fibrosis were observed in this group. In the Sham group, fibrosis (*) is observed in the papillary dermis layer. Additionally, areas of fibrosis are observed in the reticular dermis. Evidence of re-epithelialization (dashed circle) is observed in this group. In the US group, intense eosinophilic staining is observed in the collagen fibers. Basement membrane integrity is not preserved in the epithelium. In the 4-AP group, cell boundaries in the epidermis are not clearly distinguishable. The area of re-epithelialization is partially visible. In the 4-AP + US (L) group, granulation tissue (arrowhead) is noticeable. Intensely stained cellular areas in the epithelium (black arrow) indicate a proliferative effect. Fibrosis is observed in the dermis layer. Furthermore, cellular integrity (white arrow) is not preserved in the epithelium. In the 4-AP + US (M) group, dense, thick eosinophilic collagen bundles are observed. Fibrosis (*) is particularly noticeable in the dermis layer. In the 4-AP + US (H) group, granulation tissue and cellular infiltration are observed around the hair follicles in some areas. The presence of extensive fibrotic tissue is noteworthy. Staining: Hematoxylin and eosin. Scale bar = 50 µm.

In the control group, the epidermis and dermis layers maintained their normal histological integrity, and no inflammatory cell infiltration or pathological fibrosis was observed. In the Sham group, areas of significant fibrosis were detected in the papillary dermis and fibrotic foci in the reticular dermis, along with areas of re-epithelialization. In the US group, intense eosinophilic staining was noted in the collagen fibers, and the integrity of the basement membrane in the epithelium was not preserved. In the 4-AP group, epidermal cell boundaries were lost, and areas of re-epithelialization were partially visible. In the 4-AP (L) + US group, granulation tissue, densely stained cellular areas in the epithelium, dermal fibrosis, and loss of cellular integrity in the epithelium were observed. In the 4-AP (M) + US group, dense and thick eosinophilic collagen bundles and significant dermal fibrosis were detected. In the 4-AP (H) + US group, scattered granulation tissue and cellular infiltration were observed around the hair follicles, while widespread fibrotic tissue was noteworthy (Figure 1).

#### 3.1.1. Histopathological Scoring

According to the histopathological scoring results, the fibrosis score was found to be lowest in the control group. Fibrosis scores were higher in the Sham, US, 4-AP(L) + US, and 4-AP(M) + US groups, with the highest value found in the 4-AP(M) + US group. Angiogenesis scores were found to range between 0.71 and 1.86 among the groups. Granulation tissue scores were higher in the Sham and 4-AP groups and lower in the control and US groups (Table 2).

#### 3.1.2. Immunohistochemical Findings

**Figure 2:**
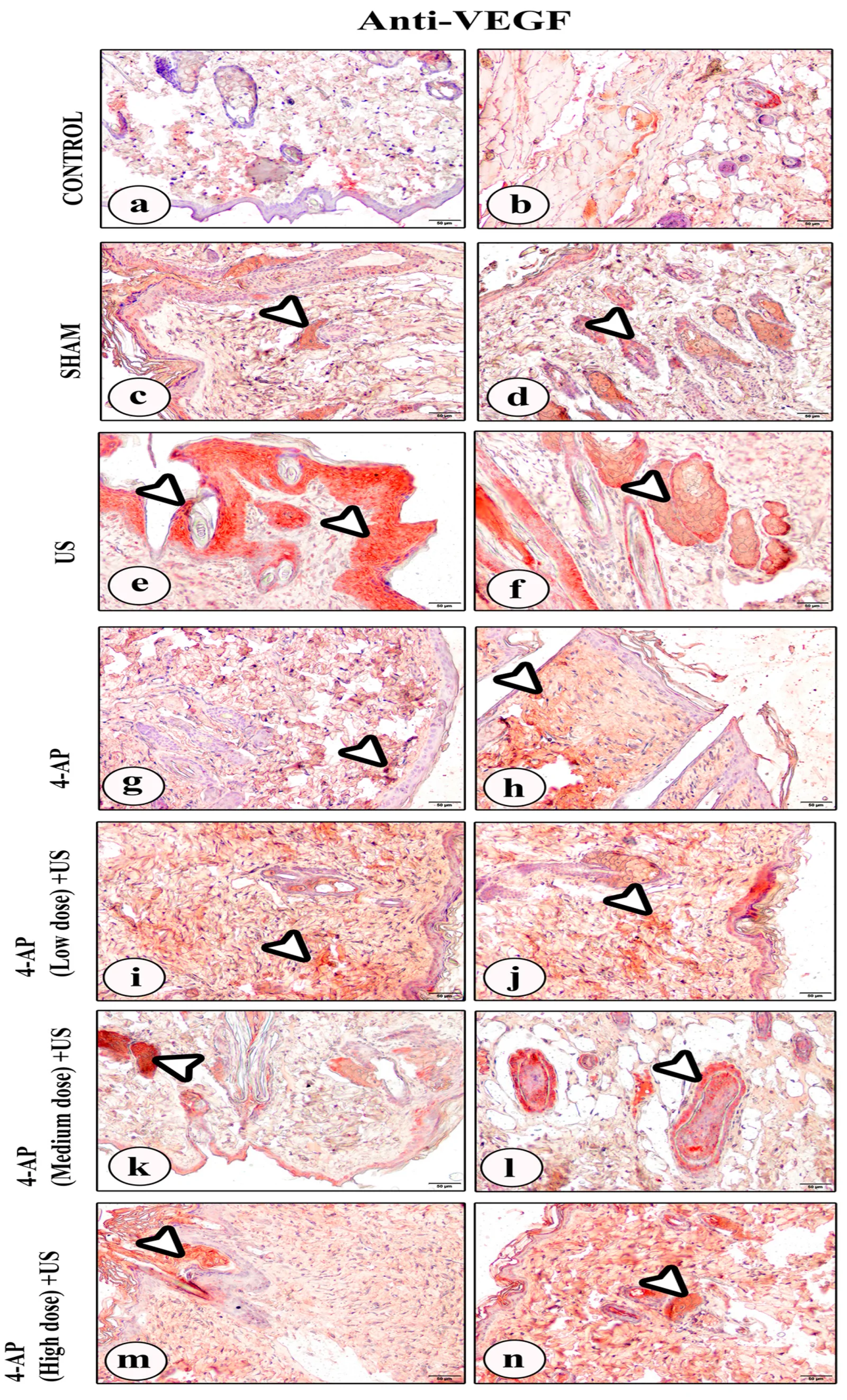
(**a**–**n**) Anti-VEGF immunoreactivity is observed in all groups. (**e**,**f**) In addition to the vascular structure, intense anti-VEGF immunoreactivity is particularly observed in the epidermal epithelium of the US group. This finding may be closely related to follicular regeneration during wound healing. Representative positively stained cells are indicated by arrowheads. Mayer’s hematoxylin was used for counterstaining. Scale bar = 50 µm.

VEGF immunoreactivity was observed in all groups. The US group had the highest VEGF immunoreactivity score. Relatively high VEGF immunoreactivity was also observed in the low-dose and high-dose 4-AP + US groups. In the morphological evaluation, significant VEGF immunopositivity was observed in the epidermal epithelium and vascular structures in the US group (Figure 2, Table 2).

**Figure 3:**
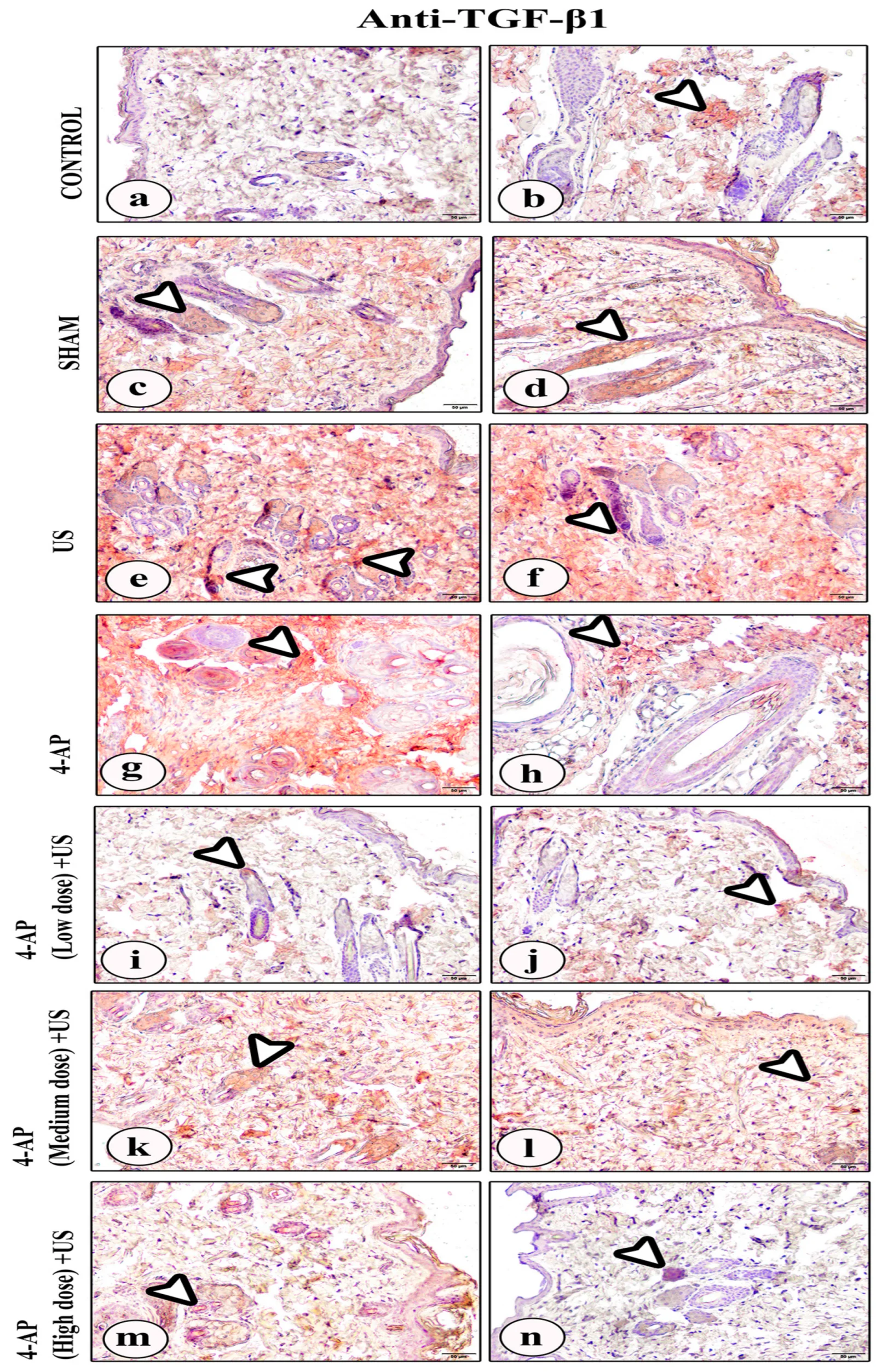
(**a**–**n**) Anti-TGF-β1 immunoreactivity is observed in all groups. High positive staining is observed in the US group. Additionally, in (**c**–**f**,**k**,**l**), positive staining is observed in the hair follicles of the Sham, US, and 4-AP (Medium dose) + US groups. Since hair follicles serve as a reservoir of epidermal stem cells, TGF-β1 may contribute to stem cell differentiation. Representative positively stained cells are indicated by arrowheads. Mayer’s hematoxylin was used for counterstaining. Scale bar = 50 µm.

**Figure 4:**
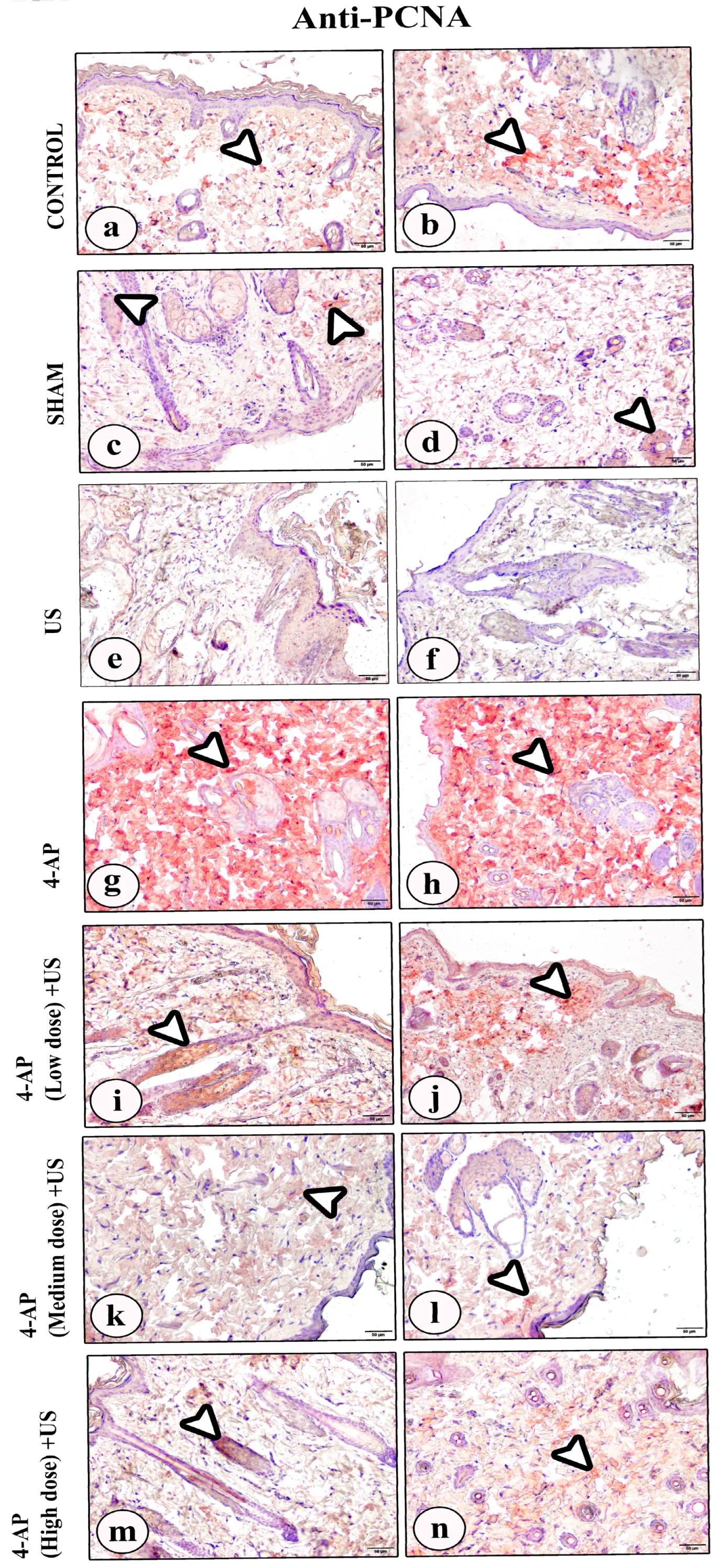
(**a**–**n**) Anti-PCNA immunoreactivity is observed in all groups. (**g**,**h**) High positive staining is observed in the 4-AP group. Anti-PCNA immunoreactivity was observed in the dermis at varying intensities in all groups, while in the 4-AP (Low dose) + US group (**i**,**j**), it was also observed in the epidermal region. Representative positively stained cells are indicated by arrowheads. Mayer’s hematoxylin was used for counterstaining. Scale bar = 50 µm.

TGF-β1 immunoreactivity was observed in all groups. A high level of positive staining was detected in the US group. In addition, TGF-β1 immunopositivity was observed around hair follicles in the Sham, US, and 4-AP(M) + US groups. According to the immunohistochemical scoring results, TGF-β1 immunoreactivity was higher in the US, 4-AP, and 4-AP(M) + US groups compared with the control group (Figure 3, Table 2). PCNA immunoreactivity was observed at varying intensities in all groups. According to the immunohistochemical scoring results, the highest PCNA immunoreactivity was observed in the 4-AP group. PCNA positivity was present in the dermis at varying intensities in all groups, while immunopositive cells were also observed in the epidermal region of the 4-AP(L) + US group. PCNA immunoreactivity was lower in the medium- and high-dose 4-AP + US groups compared with the 4-AP group (Figure 4, Table 2).

Histopathological and immunohistochemical evaluations showed different findings between the groups in terms of fibrosis, granulation tissue, and VEGF, TGF-β1, and PCNA immunoreactivity (Figure 1, Figure 2, Figure 3 and Figure 4, Table 2).

### 3.2. Molecular Findings

**Figure 5:**
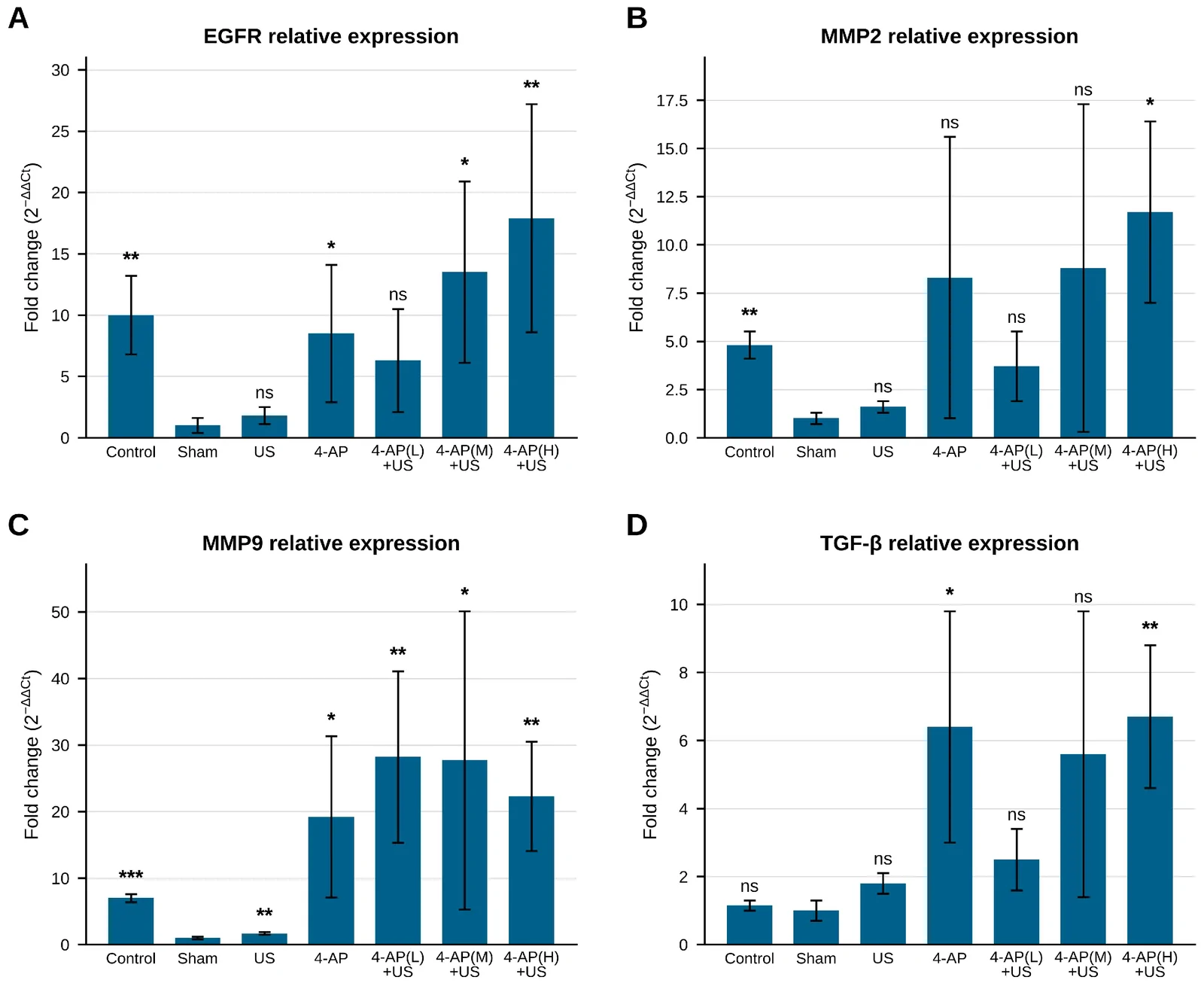
mRNA Expression Levels of Regenerative Markers (qRT-PCR). Data were normalized to the GAPDH reference gene, and the Sham group (using the 2−ΔΔCt method) was used as a calibrator. (**A**) EGFR: 4-AP(H)-US induced a strong proliferative response. (**B**) VEGFR: Angiogenic activation showed a 26.2-fold increase. (**C**) TGF-β, (**D**) MMP-2. Increases in the medium- and high-dose groups reflect extracellular matrix remodeling, tissue repair, and matrix turnover. Data are presented as the mean ± SD. * *p* < 0.05 (compared with the Sham group), ** *p* < 0.01 (compared with the US group), and *** *p* < 0.001 (indicating a statistically significant difference among the groups). ns stands for not significant.

In the molecular analyses, target gene expression levels were normalized using the GAPDH reference gene, and relative expression changes were evaluated using the ΔCt, ΔΔCt, and 2^−ΔΔCt^ methods, with the Sham group as the calibrator. The molecular markers examined included EGFR, VEGFR, TGF-β, MMP-2, and MMP-9. These genes were evaluated in relation to cellular proliferation, angiogenesis, tissue repair and fibrotic responses, and extracellular matrix remodeling, respectively. EGFR expression showed a significant increase, particularly in the groups treated with a combination of 4-AP and ultrasound, compared with the Sham group. While the increase in EGFR expression was limited in the ultrasound group, a more pronounced increase was observed in the low-dose 4-AP + US group. In the medium- and high-dose 4-AP + US groups, EGFR expression reached even higher levels. These findings suggest that the 4-AP + US combination induces a stronger proliferative response than ultrasound alone. The significant increase in EGFR, particularly in the high-dose 4-AP + US group, suggests that molecular processes associated with epidermal renewal, cell proliferation, and wound healing may be more active in this group (Figure 5).

**Figure 6:**
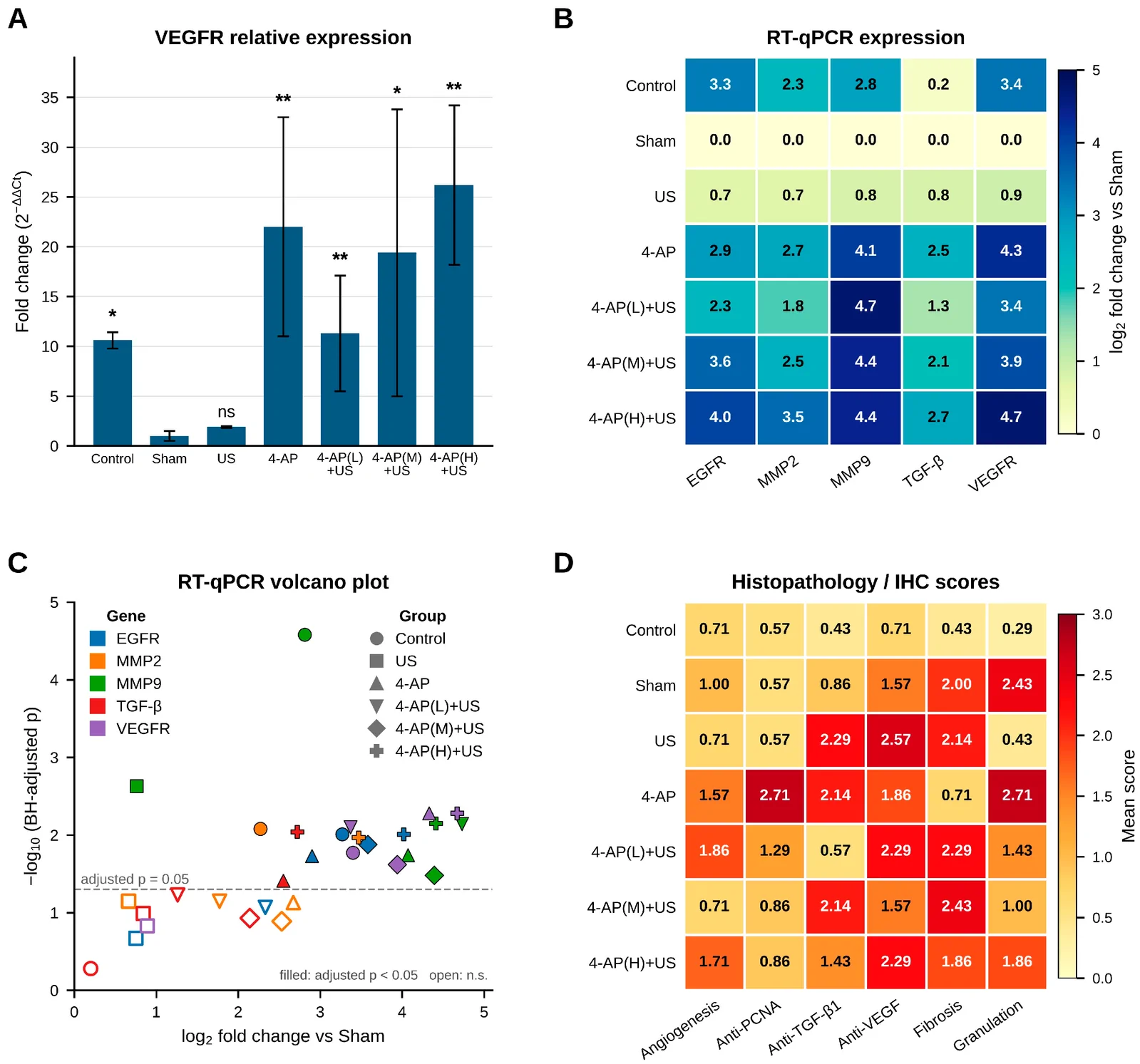
Multimodal assessment of angiogenic and tissue remodeling markers. (**A**) Normalized VEGFR mRNA expression levels (2^−ΔΔCt^) compared to the sham group. (**B**) An RT-qPCR heatmap summarizing log_2_ fold change values of target genes (EGFR, MMP2, MMP9, TGF-β, VEGFR). (**C**) A volcano plot showing the relationship between gene expression changes and statistical significance (−log_10_ *p*-value); dashed line represents significance threshold (*p* < 0.05). (**D**) Heatmap of mean histopathological/IHC scores for angiogenesis, fibrosis, and immunoreactivity parameters. Data in panel (**A**) are presented as the mean ± SD. * *p* < 0.05, ** *p* < 0.01 (compared to the sham group); ns: not significant.

When VEGFR expression was evaluated, the most significant increase was observed in the 4-AP + US combination groups. While the US group showed a slight increase compared with the Sham group, VEGFR expression increased significantly in the 4-AP(L) + US group. In the medium- and high-dose 4-AP + US groups, the increase in VEGFR expression became even more pronounced. This result suggests that the 4-AP + US combination may enhance the angiogenic response in a dose-dependent manner. Increased VEGFR expression may contribute to neovascularization, vascular remodeling, and tissue oxygenation in the wound area. The fact that VEGFR expression reached one of the highest levels, especially in the 4-AP(H) + US group, indicates that this application induces strong molecular activation of angiogenesis (Figure 6).

TGF-β expression was evaluated in terms of tissue repair, fibroblast activation, extracellular matrix production, and fibrotic responses. While a limited increase in TGF-β expression was observed in the US group, this increase remained relatively low in the 4-AP(L) + US group. In contrast, a more pronounced increase in TGF-β expression was observed in the medium- and high-dose 4-AP + US groups. These findings suggest that molecular processes associated with tissue repair and matrix organization may be more active, particularly with medium- and high-dose treatments. However, the increase in TGF-β expression should be evaluated together with the changes in MMP-9 and VEGFR expression. Rather than indicating a fibrotic response alone, this finding may reflect a response that contributes to matrix remodeling and tissue organization during the wound healing process (Figure 5).

MMP-2 expression is an important marker, particularly in relation to extracellular matrix remodeling and tissue organization. While MMP-2 expression showed a slight increase in the US group, a more pronounced increase was observed in the 4-AP + US groups. A moderate increase was observed in the low-dose group, while MMP-2 expression reached higher levels in the medium- and high-dose groups. These findings suggest that the 4-AP + US combination may support extracellular matrix remodeling during tissue healing. The increase in MMP-2, particularly in the 4-AP(H) + US group, may reflect a favorable molecular response associated with controlled matrix remodeling and organized wound healing (Figure 5).

MMP-9 expression showed one of the most pronounced changes among the target genes. The increase in MMP-9 expression was particularly evident in the low- and medium-dose 4-AP + US groups. Although MMP-9 expression was also high in the high-dose 4-AP + US group, it was relatively lower than in the low- and medium-dose groups. MMP-9 is associated with inflammatory cell migration, extracellular matrix degradation, wound clearance, and early tissue remodeling. Therefore, the high MMP-9 expression observed in the low- and medium-dose 4-AP + US groups suggests an active inflammatory remodeling and matrix turnover response. In the high-dose group, MMP-9 expression remained high but was accompanied by increases in VEGFR, EGFR, and MMP-2 expression. This may indicate that, in addition to inflammatory remodeling, a more organized regenerative response may have developed in this group (Figure 5).

In the molecular profile evaluation results of the 4-aminopyridine group, a significant increase in EGFR, VEGFR, MMP-2, MMP-9, and TGF-β expression was observed compared to the Sham group. This suggests that 4-aminopyridine application alone may have a stimulating effect on cellular proliferation, angiogenesis, and matrix remodeling. However, in the 4-AP + US combination groups, especially at medium and high doses, this molecular response became more pronounced, suggesting that the combination application may have a synergistic or potentiating effect (Table 3).

When the ultrasound group was evaluated molecularly, a slight increase was observed in all the genes examined compared to the Sham group. However, these increases remained limited compared to the 4-AP + US combination groups. This finding suggests that ultrasound application alone causes a low level of biological activation, but this effect is significantly enhanced when combined with 4-aminopyridine (Figure 5).

The Control group showed a higher basal expression profile in some genes compared to the Sham group. This situation may be related to the maintenance of physiological cellular regeneration, vascular homeostasis, and basal matrix turnover in healthy tissue. Because the Sham group was a surgical/procedural control, differences between the Control and Sham groups should be interpreted in terms of tissue integrity, trauma response, and basal healing processes (Figure 6).

A general molecular assessment reveals that the 4-AP + US combination significantly increases VEGFR, EGFR, and MMP2 expression, supporting a robust regenerative response associated with angiogenesis, cellular proliferation, and organized extracellular matrix remodeling. The marked increase in MMP9 expression suggests that inflammatory remodeling and matrix turnover processes are active, particularly in the low and medium-dose 4-AP + US groups. The significant increase in VEGFR, EGFR, MMP2, and TGF-β expression in the 4-AP (H) + US group indicates that this group not only exhibits an inflammatory response but also a more organized and regenerative healing profile. Therefore, the molecular findings demonstrate that the combination of 4-aminopyridine and ultrasound activates key molecular pathways associated with wound healing and tissue regeneration, that this effect is stronger than that of ultrasound application alone, and that a more pronounced regenerative molecular profile emerges, especially with the high-dose combination treatment (Figure 5 and Figure 6 and Table 3).

### 3.3. Morphometric Findings

The method used for the morphological evaluation of the wound area in this study was as follows: According to the simple ruler method, the surface area was determined by multiplying the length and width of the wound. To assess the third dimension of the wound, wound thickness was measured in the isolated skin using an electronic caliper. Thus, mass, area, and volume measurements were used to determine the morphological characteristics of the wound (Table 4).

## 4. Conclusions

Restoration of skin integrity is a complex biological process involving the active participation of various cell types, starting with hemostasis and progressing to tissue remodeling [1,4]. In cases of skin damage, such as deep dermal injuries and burns, where skin integrity is compromised, overcoming the SC, the skin’s natural barrier, and delivering regenerative signals to deeper tissues remain clinical challenges [3].

Particularly in cases of deeper skin injuries, the natural barrier formed by the stratum corneum can limit the adequate delivery of topically applied therapeutic agents to the target tissues. This situation highlights the importance of transdermal delivery approaches that aim to deliver therapeutic agents capable of supporting wound healing directly to the target tissue, particularly in anatomical areas such as the face, where aesthetic concerns are paramount. Furthermore, evaluating treatment responses related to wound healing in the face, an anatomically and aesthetically important region, represents a clinically meaningful area of research. Therefore, in the present study, a primary sutured acute wound model was created in the facial region to evaluate treatment response in an anatomically visible area.

In addition, ultrasound-mediated phonophoresis has been investigated as a potential approach to enhance the local delivery of the therapeutic agent under study and promote its effects during the wound healing process. In this context, ultrasound-assisted phonophoresis is a biophysical method that can temporarily increase skin permeability and facilitate the transdermal delivery of topical agents [5,14].

In this study, the effects of topical application of 4-aminopyridine (4-AP), a potassium channel blocker, and its administration via ultrasound-mediated phonophoresis on the healing process of sutured skin wounds were evaluated using histopathological, immunohistochemical, molecular, and morphometric parameters. The current literature indicates that phonophoresis can alter the barrier properties of the stratum corneum through cavitation and associated transient transport pathways, thereby enhancing the transdermal transport of various molecules [12,15,16]. Previous studies have shown that 4-AP may support re-epithelialization, cellular proliferation, angiogenesis, and tissue regeneration in various experimental skin injury models [3,4]. In this study, the effects of phonophoresis were evaluated based on tissue responses observed at the histopathological, immunohistochemical, molecular, and morphometric levels, rather than through direct pharmacokinetic measurements. The significant biological changes observed in the 4-AP + US groups suggest that the application of 4-AP via ultrasound-mediated phonophoresis may modulate the biological response in wound tissue. However, the findings of the present study should not be interpreted as direct evidence of increased transdermal penetration of 4-AP. The observed wound healing response may have resulted from increased tissue penetration of 4-AP, from the independent biological effects of ultrasound, or from a combination of these effects; distinguishing among these possibilities is beyond the scope of the current experimental design. Therefore, future studies that directly and quantitatively assess transdermal 4-AP penetration may help elucidate the contribution of phonophoresis to local drug delivery and provide more detailed information about the underlying mechanisms.

Several studies on wound healing have shown that low-frequency ultrasound applications generate subcutaneous cavitation dynamics through ultrasound-induced mechanical energy [20,21]. Previous experimental studies examining the effects of 4-AP on wound healing suggest that this molecule may support processes involved in cellular proliferation and tissue regeneration. 4-AP enhanced skin wound healing in a full-thickness excisional wound model by promoting NGF-associated regenerative responses, keratinocyte proliferation and migration, re-epithelialization, hair follicle neogenesis, angiogenesis, myofibroblast differentiation, and reinnervation [4]. More recently demonstrated that 4-AP promotes angiogenesis, keratinocyte proliferation and differentiation, and epidermal and dermal regeneration in a burn wound model established in mice [3]. Taken together, these findings suggest that 4-AP may influence numerous cellular and molecular processes involved in skin repair and tissue regeneration [3,4].

In the present study, the highest level of PCNA immunoreactivity was observed in the 4-AP group among all groups, supporting the association between 4-AP treatment and the proliferative cellular response and generally consistent with previous experimental findings. Similarly, the increase in EGFR expression following 4-AP administration, reaching its highest level (17.9-fold) in the 4-AP(H) + US group, suggests that the combination therapy may more strongly influence molecular signaling processes associated with proliferation and epidermal renewal. The central role of growth factors in wound healing has been extensively documented, and studies have reported that the EGF family plays important roles in regulating keratinocyte proliferation, migration, and epidermal repair [22,23]. Based on this information, the increase in EGFR observed in our study can be interpreted as an indicator of the molecular response associated with the proliferative phase of wound healing. However, although PCNA immunoreactivity was highest in the 4-AP group, EGFR expression reached its highest level in the 4-AP(H) + US group, indicating that these two markers did not show parallel changes. This difference may be related to PCNA and EGFR reflecting different components of the wound healing process.

While PCNA reflects cellular proliferative activity, EGFR expression may reflect changes associated with growth factor-mediated signaling. Therefore, the lack of a completely parallel pattern between the two parameters across the dose groups can be interpreted not as an inconsistency, but rather as a complementary finding reflecting different cellular and molecular aspects of wound healing. Thus, the combined evaluation of the histological and molecular findings suggests that 4-AP alone induces a marked proliferative response, whereas its combination with ultrasound may modulate the EGFR-related molecular response to a greater extent. One of the possible biological mechanisms underlying this effect may be the effect of 4-AP on voltage-gated potassium channels, as 4-AP has been reported to prolong membrane depolarization by reducing the outward K^+^ current and, as a result, may affect voltage-gated Ca^2+^ channels and calcium-dependent cellular signaling mechanisms [24]. It is known that these changes in intracellular Ca^2+^ signaling can modulate signaling pathways associated with gene transcription, cellular proliferation, and tissue repair [25,26]. This mechanistic framework may provide a biologically plausible explanation for the changes in PCNA immunoreactivity and EGFR expression observed in our study.

Accordingly, the current findings suggest that 4-AP may influence the proliferative components of wound healing through cellular signaling associated with ion channel modulation, and that its administration in combination with ultrasound may alter the magnitude of this molecular response.

The detailed intracellular mechanisms underlying this relationship could be further elucidated in future studies that directly evaluate Kv channel activity and Ca^2+^-dependent signaling mechanisms.

Regarding angiogenesis, our findings are generally consistent with previous experimental studies reporting angiogenesis-promoting effects of 4-AP during wound healing. 4-AP administration increased angiogenesis while reducing inflammation and apoptosis in an experimental burn model [3], whereas reported that 4-AP treatment was associated with increased angiogenesis alongside improved skin wound healing and tissue regeneration in a full-thickness excisional wound model [4]. These findings provide experimental support for a potential role of 4-AP in promoting angiogenic responses during skin repair [4].

Similarly, in the present study, VEGFR expression increased 22.0-fold in the 4-AP group, 19.5-fold in the 4-AP(M) + US group, and 26.2-fold in the 4-AP(H) + US group, with the highest VEGFR response observed in the 4-AP(H) + US group. These findings suggest that, particularly when combined with ultrasound, 4-AP may more strongly modulate the angiogenesis-related molecular response and are consistent with previous experimental findings in the context of the sutured wound model.

The immunohistochemical findings are consistent with the results obtained from the molecular analyses. Although VEGF immunoreactivity was observed in all groups, the highest score was detected in the US group, while marked VEGF positivity was also observed in the low- and high-dose 4-AP + US groups. VEGF is one of the principal proangiogenic growth factors involved in angiogenesis during cutaneous wound healing [27,28].

In contrast, VEGFR expression was highest in the 4-AP(H) + US group, indicating that the VEGF and VEGFR responses did not follow completely parallel patterns across the groups. The VEGF family comprises growth factors that play central roles in angiogenesis, whereas VEGFRs are key components of the receptor system that mediates the cellular effects of these ligands [29]. Therefore, the differential patterns of VEGF immunoreactivity and VEGFR expression may reflect the regulation of angiogenesis-related responses at distinct biological levels. In this context, the strong VEGF immunoreactivity observed in the US group suggests that ultrasound alone may influence the local angiogenesis-related response, whereas the marked increase in VEGFR expression in the 4-AP + US groups, particularly in the 4-AP(H) + US group, suggests that the combined application of 4-AP and ultrasound may further modulate the angiogenesis-related molecular response. The combined evaluation of histological and molecular findings constitutes a significant strength of the present study, providing complementary evidence regarding angiogenesis-related responses.

The different patterns observed in VEGF immunoreactivity and VEGFR expression may reflect a multilayered regulation of angiogenesis at the ligand, receptor, and tissue levels, rather than a direct discrepancy between these findings. In this context, our results suggest that 4-AP and ultrasound application may modulate angiogenesis-related biological responses at different regulatory levels. When considered together with previous studies showing that 4-AP supports angiogenesis and enhances VEGF-related responses during cutaneous wound healing, the current findings further support the potential role of 4-AP in the angiogenic component of wound healing [3,4]. Furthermore, the more pronounced VEGFR expression observed in the 4-AP(H) + US group suggests that the combination of 4-AP with ultrasound-mediated phonophoresis may be associated with differential modulation of angiogenesis-related molecular responses [3,4].

During wound healing, particularly during the process of tissue remodeling, matrix metalloproteinases (MMPs) are among the primary proteolytic systems involved in the removal and reorganization of the damaged extracellular matrix, cell migration, and matrix remodeling. Toriseva and Kähäri (2009) emphasized that MMPs play a fundamental role in regulating matrix turnover and cellular motility during cutaneous wound healing; however, they also noted that their proteolytic activities must be tightly regulated in terms of both timing and level [30]. In the present study, the 28.2- and 27.7-fold increases in MMP9 expression in the 4-AP(L) + US and 4-AP(M) + US groups, respectively, may indicate that enhanced extracellular matrix turnover became more pronounced in response to low- and medium-dose combination therapies. The lower 22.3-fold increase observed in the high-dose group, however, indicates that this response does not increase linearly with the rise in 4-AP dose. Therefore, it seems more appropriate to interpret the increase in MMP9 expression not merely as an indicator of improved wound healing, but as a molecular finding associated with active extracellular matrix turnover and tissue reorganization within the wound [30].

The interpretation of these molecular findings together with the histopathological results provides further insight into the tissue-level response. In the medium-dose 4-AP + US group, dense and thick eosinophilic collagen bundles and marked dermal fibrosis were observed, with the highest fibrosis score recorded in this group. In the high-dose group, granulation tissue, cellular infiltration around hair follicles, and prominent fibrotic tissue were also observed. Taken together, these histopathological findings provide tissue-level evidence consistent with active matrix remodeling and reorganization, which may be related to the elevated MMP9 expression observed in the 4-AP + US groups.

Given that MMP activity during wound healing is both necessary and tightly regulated, the differential MMP9 responses across the dose groups may reflect variations in the intensity and organization of extracellular matrix remodeling rather than a simple linear relationship between 4-AP dose and healing response [30]. In this context, the higher MMP9 expression observed with low- and medium-dose 4-AP + US treatment, together with the corresponding histopathological findings, may indicate more active extracellular matrix turnover and tissue reorganization, whereas the lower response at the high dose suggests that increasing the 4-AP dose does not necessarily result in a proportionally greater remodeling response.

The findings regarding MMP2 and TGF-β also point to a different aspect of matrix remodeling. MMP2 is an important gelatinase associated with extracellular matrix turnover and tissue remodeling during wound healing [31]. MMP2 expression reached its highest level, with an 11.7-fold increase, in the 4-AP(H) + US group, while TGF-β expression increased 6.8-fold in the same group; in the medium-dose combination group, MMP2 and TGF-β expression increased 8.8-fold and 5.6-fold, respectively. This molecular profile suggests that tissue responses related to matrix organization and fibroblast activity became more pronounced, particularly in the medium- and high-dose combination groups. The role of TGF-β signaling in regulating fibroblast activation, extracellular matrix accumulation, and fibrotic responses supports this interpretation [32,33]. However, it may not be appropriate to evaluate this response solely in terms of regeneration, as collagen deposition and fibroblast-related responses are important components of the wound healing process and may vary depending on the stage and context of healing [30,32,33]. In the present study, the histopathological increase in collagen density and fibrotic changes observed in the same groups suggest that fibrotic responses may progress simultaneously with matrix remodeling [34].

The reported findings regarding the potential relationship between ion channel modulation and the matrix response are also noteworthy [13]. In this study, it was shown that collagen expression in human dermal fibroblasts is associated with Kv1.3 channels. Although direct measurement of Kv1.3 activity or collagen gene expression was not performed in the current study, the changes observed in collagen density, fibrosis, and matrix remodeling after 4-AP administration suggest a potential biological relationship between ion channel modulation and fibroblast and matrix responses. This potential relationship may be elucidated in more detail through further studies evaluating both Kv1.3 channel activity and fibroblast/matrix responses.

These findings indicate that 4-AP + US treatment significantly influences responses associated with extracellular matrix remodeling; however, this effect may result in different balances among MMP9-associated matrix turnover, MMP2/TGF-β-associated matrix organization, and the fibrotic response at different doses. Therefore, one of the significant contributions of this study is that, rather than interpreting healing based solely on changes in a single molecular marker, it provides a more comprehensive assessment of matrix remodeling by evaluating molecular and histopathological findings together.

Hormetic and biphasic dose-response models have been demonstrated in epidermal stem cells, and it has been emphasized that such non-linear dose-response models may also be observed in fibroblast-mediated wound healing responses [34,35]. Similarly, in the present study, some of the strongest molecular responses in terms of EGFR, VEGFR, TGF-β, and MMP2 were observed in the 4-AP(H) + US group, while fibrotic changes and high tissue volume persisted in the same group. The fact that the highest histopathological fibrosis score accompanied significant molecular activation in the medium-dose group further indicates that the wound healing response cannot be defined solely by molecular expression levels.

These findings indicate that different doses can affect different components of wound healing, such as proliferation, angiogenesis, and matrix remodeling, to varying degrees. Therefore, the current findings suggest that 4-AP + US administration generates a multidimensional biological response profile rather than a simple linear dose–response relationship. This perspective allows the prominence of different doses at different endpoints to be interpreted not as an inconsistency, but as an indication that the treatment may modulate different components of wound healing to different extents. However, the observed nonlinear profile should not be interpreted as direct evidence that phonophoresis increases 4-AP penetration; rather, the current data indicate that the combination treatment produces non-monotonic responses across different biological endpoints.

Morphometric assessments further support the multidimensional nature of the molecular and histological findings. Among the combination groups, skin thickness closest to the control group value was observed in the 4-AP(L) + US group, tissue volume in the 4-AP(M) + US group, and wound area in the 4-AP(H) + US group. This distribution suggests that different doses may affect different structural components of wound healing at different levels. Although a significant difference was observed between the groups in terms of skin thickness, the fact that the combination groups shared the same letter groupings as the control and Sham groups in the post hoc analysis suggests that the treatment may have modulated tissue thickness rather than indicating that a specific dose was superior. In contrast, the lack of statistically significant differences in wound area suggests that surface area alone may have limited value in distinguishing the healing response. It has been reported that area and volume measurements in wound assessment enable the evaluation of tissue changes across different dimensions, and that three-dimensional volume measurements can provide additional information regarding wound depth [19]. In this respect, the current findings support the importance of evaluating wound healing not only in terms of wound closure but also in conjunction with different morphometric parameters such as tissue thickness and volume.

The regenerative effects of 4-AP on skin wound healing have been demonstrated previously in various experimental models. The significant changes in molecular and immunohistochemical markers associated with proliferation and angiogenesis detected in the present study are generally consistent with the literature. However, unlike previous studies, this study primarily evaluated a sutured acute postoperative wound model in the facial region. This difference in the experimental model is an important factor that may explain why the PCNA–EGFR and VEGF–VEGFR responses were not perfectly parallel across all dose groups and why fibrotic remodeling was more pronounced at medium and high doses. Therefore, these results suggest that the effect of 4-AP on wound healing may result in different biological profiles depending on the wound type, anatomical region, and tissue microenvironment. Since previous studies focused on full-thickness dorsal wound and burn models, the data obtained in the present sutured postoperative wound model provide a complementary contribution to the literature.

A similar pattern was observed regarding ultrasound.

It has been reported that low-frequency ultrasound application may have beneficial effects on the healing and closure of chronic lower-extremity wounds [20]. Furthermore, debridement using low-frequency ultrasound has been evaluated in patients with diabetic foot ulcers and osteomyelitis. In this study, the marked VEGF and TGF-β1 immunoreactivity observed in the ultrasound group suggests that ultrasound application alone can modulate the local tissue response [21]. In contrast, gene expression changes assessed by qRT-PCR were more limited in the US group, whereas responses involving EGFR, VEGFR, MMP2, MMP9, and TGF-β were particularly pronounced in the combination groups. These findings suggest that the presence of 4-AP may alter the nature and magnitude of the biological response.

When all findings are evaluated together,4-AP alone was not sufficiently effective. Indeed, PCNA immunoreactivity was found to be at its highest level in the 4-AP group, and significant increases in the expression of EGFR, VEGFR, TGF-β, MMP2, and MMP9 were observed compared with the Sham group.

In the combination groups, the fact that some molecular markers reached higher levels suggests that the 4-AP + US combination may be associated with the emergence of responses related to proliferation, angiogenesis, and matrix remodeling at varying levels. These findings suggest that the combined application of pharmacological and biophysical components may elicit a more pronounced response profile at different biological endpoints in wound tissue.

Overall, the present study presents a comprehensive approach that supports the previously reported proliferative, angiogenic, and regenerative effects of 4-AP in a sutured postoperative wound model and evaluates these effects collectively at the histopathological, immunohistochemical, molecular, and morphometric levels. At the same time, the prominence of different endpoints at different doses indicates that the 4-AP + US response is not unidirectional but rather multifaceted. In particular, the fact that fibrotic remodeling accompanies strong molecular activation at medium and high doses highlights that therapeutic efficacy should be evaluated not only based on increases in gene expression but also on the balance among proliferation, angiogenesis, and extracellular matrix organization. In this regard, this study provides detailed experimental data on the biological effects of the 4-AP + US combination on suture wound healing and lays the groundwork for further studies aimed at determining the appropriate dose range.

This study presents a comprehensive experimental approach that evaluates the effects of a combination of 4-AP and phonophoresis on suture wound healing at histopathological, immunohistochemical, molecular, and morphometric levels. However, several limitations should be considered when interpreting the findings.

Firstly, although the ability of phonophoresis to enhance transdermal drug transport has been well documented in the literature, 4-AP concentrations and transdermal 4-AP transport were not directly evaluated using pharmacokinetic methods in the present study. Therefore, while the significant biological responses observed in the combination groups demonstrate that ultrasound-guided 4-AP application can modulate tissue responses, they do not directly establish the quantitative extent of transdermal 4-AP transport or demonstrate that these responses resulted from increased 4-AP penetration. Furthermore, because ultrasound alone can affect several biological parameters, the specific contribution of ultrasound to the responses observed in the combination groups cannot be completely separated from the effects of 4-AP using the current experimental design. In future studies, direct assessment of tissue 4-AP concentrations and local pharmacokinetic parameters may help elucidate the contribution of phonophoresis to local drug delivery in greater detail.

Secondly, because the wound model was created in the facial region, the size of the tissue samples obtained was insufficient for biomechanical assessments such as tensile strength testing. Therefore, although the structural and molecular components of wound healing were evaluated in detail, mechanical tissue strength was not assessed.

Thirdly, because this study was conducted in an experimental animal model, the findings cannot be directly generalized to human wound healing or clinical practice. In addition, the relatively short follow-up period did not allow for the evaluation of longer-term changes, such as late-stage tissue remodeling and scar maturation. Therefore, the translational relevance of the findings should be further investigated in studies incorporating longer follow-up periods.

Fourthly, the combined assessment of EGFR, VEGFR, TGF-β, MMP2, and MMP9 expression with PCNA, VEGF, and TGF-β1 immunoreactivity provided a multilayered evaluation of proliferation, angiogenesis, and matrix remodeling during wound healing. However, further studies evaluating Kv channel activity, Ca^2+^-dependent signaling mechanisms, and related protein levels may help clarify the underlying mechanisms and potential causal relationships between these pathways.

Finally, the varying molecular, histopathological, and morphometric responses observed at different doses suggest that 4-AP + US administration elicits a dose-sensitive and multidimensional response. However, the absence of a monotonic and proportional response across doses limits the identification of a single dose as “optimal” for all endpoints and does not allow a direct dose–penetration relationship to be established from the available data. Therefore, the therapeutic dose range should be investigated further using broader dose ranges and multiple follow-up time points. Future studies incorporating direct measurement of tissue 4-AP levels, vascular density, biomechanical properties, and different stages of wound healing may provide a more comprehensive understanding of the mechanistic and translational relevance of the present findings.

## Figures and Tables

**Table 1:** Primer Sequences Used in qRT-PCR Analysis.

Gene/Protein	Direction	Direction Primer Sequence (5′→3′)	Product Size (bp)
**EGFR**	**F** **R**	TGC CAA AGG CAC GAG TAA CA CAG GGC ACG GTC ACC ATA AC	~113 bp
**VEGFR2/KDR**	**F** **R**	GGA ACG TAC TGG TGA AAC CGG CGA GTG AGT GTT GAG TGC AT	~164 bp
**TGF-** **beta1**	**F** **R**	TAC CTG AAC CCG TGT TGC TC GTT GCT GAG GTA TCG CCA GG	~152 bp
**MMP2**	**F** **R**	TAC ACC AAG AAC TTC CGC CTG GTC AGG ATC AGC GGC TTA CG	~144 bp
**MMP9**	**F** **R**	TGA ACC AGC ACC ACA ACA AC GAA ACC GGT TGC CTT CGA AG	~165 bp
**GAPDH**	**F** **R**	AAT CCC ATC ACC ATC TTC CAG AAA TGA GCC CCA AGC CTC AC	~120 bp

**Table 2:** Histopathological and immunohistochemical scoring.

	CONTROL	SHAM	US	4-AP	4-AP(L)-US	4-AP(M)-US	4-AP (H)-US
**Fibrosis**	0.43 ± 0.53 ^*,**,#^	2 ± 0.58	2.14 ± 0.69 ^*^	0.71 ± 0.76 ^&^	2.29 ± 0.53 ^#^	2.43 ± 0.53 ^**,&^	1.86 ± 0.69
**Angiogenesis**	0.71 ± 0.76	1 ± 0.58	0.71 ± 0.76	1.57 ± 0.53	1.86 ± 1.07	0.71 ± 0.76	1.71 ± 0.76
**Granulation**	0.29 ± 0.49 ^*,***^	2.43 ± 0.53 ^*,#^	0.43 ± 0.53 ^#,^^^	2.71 ± 0.49 ^***,^^^	1.43 ± 1.13	1 ± 0.58	1.86 ± 0.69
**Anti-VEGF immunoreactivity**	0.71 ± 0.49 ^**,*,#^	1.57 ± 0.53	2.57 ± 0.53 ^**^	1.86 ± 0.69	2.29 ± 0.76 ^*^	1.57 ± 0.98	2.29 ± 0.76 ^#^
**Anti-TGF-β1 immunoreactivity**	0.43 ± 0.53 ^**,*,#^	0.86 ± 0.69	2.29 ± 0.49 ^**,##^	2.14 ± 0.69 ^*,^^	0.57 ± 0.53 ^##,^,&^	2.14 ± 0.69 ^#,&^	1.43 ± 0.53
**Anti-PCNA immunoreactivity**	0.57 ± 0.53 ^**^	0.57 ± 0.53 ^^^^	0.57 ± 0.79 ^##^	2.7 ± 0.49 ^**,^^,##^	1.29 ± 0.95	0.86 ± 0.69	0.86 ± 0.69

^*,#,^,&^: *p* < 0.05; ^**,##,^^^: *p* < 0.01; ^***^: *p* < 0.001. The same symbols in each row represent the difference between the two groups.

**Table 3:** Statistical Evaluation of RT-qPCR Molecular Findings.

Gen	Control (S)	Sham	US	4-AP	4-AP(L) + US	4-AP(M) + US	4-AP(H) + US	*p*
EGFR (Fold Change)	10.0 ± 2.1 ***	1.0 ± 0.2	1.8 ± 0.4	8.5 ± 1.7 **	6.3 ± 1.2 *	13.5 ± 2.8 ***	17.9 ± 3.1 ***	<0.001
VEGFR (Fold Change)	10.6 ± 2.4 ***	1.0 ± 0.3	1.9 ± 0.5	22.0 ± 3.7 ***	11.3 ± 2.1 **	19.5 ± 3.4 ***	26.2 ± 4.1 ***	<0.001
TGF-β (Fold Change)	1.15 ± 0.3	1.0 ± 0.2	1.8 ± 0.4	6.4 ± 1.5 **	2.5 ± 0.7 *	5.6 ± 1.2 **	6.8 ± 1.4 ***	<0.01
MMP2 (Fold Change)	4.8 ± 1.1 *	1.0 ± 0.2	1.6 ± 0.3	8.3 ± 1.8 **	3.7 ± 0.9 *	8.8 ± 1.7 **	11.7 ± 2.6 ***	<0.001
MMP9 (Fold Change)	7.0 ± 1.5 **	1.0 ± 0.3	1.7 ± 0.4	19.2 ± 3.2 ***	28.2 ± 5.1 ***	27.7 ± 4.8 ***	22.3 ± 3.9 ***	<0.001

In the analysis of RT-qPCR data, GraphPad Prism 10.2 software was used. The assumption of normal distribution was evaluated. One-way analysis of variance (ANOVA) was used for intergroup comparisons. Tukey’s post hoc test was applied for multiple group comparisons. Data are presented as mean ± SD. Fold change values were calculated using the 2^−ΔΔCt^ method, with GAPDH as the reference gene and the Sham group as the calibrator. The *p* values in the last column were obtained by one-way ANOVA. Asterisks indicate significance compared with the Sham group according to [Dunnett’s/Tukey’s] post hoc test: * *p* < 0.05, ** *p* < 0.01, *** *p* < 0.001.

**Table 4:** Morphometric evaluation of skin tissue among experimental groups.

Groups	Thickness (mm)	Mass (g)	Volume (mm^3^)	Area (mm^2^)
Control	1.184 ± 0.225 ^a^	0.214 ± 0.069	134.9 ± 31.4	116.333 ± 31.758
Sham	2.116 ± 0.367 ^b^	0.260 ± 0.055	230.6 ± 44.2	107.092 ± 111.589
US	1.944 ± 0.207 ^ab^	0.283 ± 0.075	216.3 ± 88.2	100.537 ± 33.539
4-AP	2.118 ± 0.433 ^b^	0.240 ± 0.055	217.6 ± 42.9	112.812 ± 24.170
4-AP(L) + US	1.552 ± 0.334 ^ab^	0.220 ± 0.045	172.8 ± 56.8	110.078 ± 20.270
4-AP(M) + US	1.838 ± 0.476 ^ab^	0.220 ± 0.045	154.5 ± 47.9	83.798 ± 9.885
4-AP(H) + US	1.812 ± 0.362 ^ab^	0.267 ± 0.121	220.9 ± 87.3	118.895 ± 31.770

Values are expressed as the mean ± standard deviation (SD). Different superscript letters indicate statistically significant differences between groups for skin thickness (Bonferroni post hoc test, adjusted significance level *p* < 0.0024). Groups sharing the same superscript letter are not significantly different.

## Data Availability

The original contributions presented in this study are included in the article. Further inquiries can be directed to the corresponding author.

## Abbreviations

The following abbreviations are used in this manuscript:

4-AP	4-Aminopyridine (selective potassium (K^+^) channel blocker)
EGFR	Epidermal Growth Factor Receptor
VEGFR	Vascular Endothelial Growth Factor Receptor
VEGF	Vascular Endothelial Growth Factor
MMPs	matrix metalloproteinases
MMP2	Matrix Metalloproteinase-2
MMP9	Matrix Metalloproteinase-9
TGF-β/TGF-β1	Transforming Growth Factor Beta
PCNA	Proliferating Cell Nuclear Antigen
NGF	Nerve Growth Factor
BDNF	Brain-Derived Neurotrophic Factor
GAPDH	Glyceraldehyde-3-Phosphate Dehydrogenase (housekeeping/reference gene)
eNOS	Endothelial Nitric Oxide Synthase
p75-NTR	p75 Neurotrophin Receptor
SOX10	SRY-Box Transcription Factor 10 Methodological and Experimental Terms
US	Ultrasound
DMSO	Dimethyl Sulfoxide (solvent used in hydrogel preparation)
SC	Stratum Corneum (outermost layer of the skin barrier)
qRT-PCR	Quantitative Real-Time Polymerase Chain Reaction
IHC	Immunohistochemistry
L	Low-Dose
M	Medium-Dose
H	High-Dose
Vm	Resting Membrane Potential
Ca^2+^	Calcium Ion
K^+^	Potassium Ion
